# Editorial: International Partnerships for Strengthening Health Care Workforce Capacity: Models of Collaborative Education Part II

**DOI:** 10.3389/fpubh.2018.00152

**Published:** 2018-05-23

**Authors:** Jeanne M. Leffers

**Affiliations:** Community Nursing, University of Massachusetts Dartmouth, North Dartmouth, MA, United States

**Keywords:** partnerships, collaboration, education, sustainability, health workforce capacity, global health

Part II of the e-book, *International Partnerships for Strengthening Health Care Workforce Capacity: Models of Collaborative Education*, offers significant examples of key elements for international collaborative education to build health workforce capacity to improve health care outcomes, and provide guidance for building or maintaining partnerships. While Part I includes 18 manuscripts that focus extensively on long term partnership development or programs, Part II offers 13 more examples of those partnership elements or short term educational programs. As noted in the Editorial by Leffers and Audette that precedes Part I of this e-book, the manuscripts represent geographic and professional diversity and range from those that address partnership elements, program development, multifaceted offerings of university partnerships, and short-term educational offerings to strengthen health care workforce capacity.

The entire collection of 31 published manuscripts from the Research Topic (RT) represent perspectives, community case study, curriculum, instruction and pedagogy, and evaluation types across academic, non-governmental organization and other global partnership forms. While every manuscript included in this RT addresses partnerships, the 18 manuscripts published as Part I of this e-book strongly exemplify partnership elements of collaboration, mutual planning, and capacity building (1–3) while those in Part II are more specific to programs and short term educational offerings.

In Part II of this e-book are 13 manuscripts that offer examples of comprehensive assessment for collaborative program planning, program development, effective short-term educational offerings, and academic partnerships with multifaceted activities with several global host settings. While these 13 manuscripts do not address all partnership and sustainability aspects of the RT for long-term programs that are described in Part I, they do address important elements of partnerships that address workforce capacity. Part II offerings address projects that focus upon both health and partner priorities such as capacity building for midwives, occupational and physical therapists, nurses, anesthesiologists, and nurse anesthetists and medical specialties in trauma, orthopedics, and infectious disease. Collaboration, partnership building and meeting host setting needs feature strongly in the manuscripts in both Part I and Part II of this e-book. Canizares et al. discuss an extensive needs assessment to build health promotion programs for children with upper limb differences that extended on-going work in a long-term partnership. The assessment can serve as a model of stakeholder engagement that looked at a wide range of factors such as demographics, environment, socioeconomics, individual behaviors, and biologic factors to discover the most important priorities for families [1]. Five of the manuscripts in Part II highlight program development: Cunningham et al. for a physical therapy program in Kenya, Persaud et al. for an infectious disease program in Guyana, Manske for a pediatrics hand and upper extremity surgical program in Nicaragua, while Potisek et al. conducted a survey of 93 graduates of a nurse anesthesia training school in Cunningham and McFelea  and McFelea assess knowledge, clinical reasoning, and psychomotor skills for physical therapists in a post-graduate orthopedic manual therapy program. Two authors describe university partnerships with multifaceted offerings. Brzoska et al. highlight the International Public Health partnership between a university in Germany and universities in India, Nigeria, and Turkey. Conway et al. explain the four strategic areas for the Institute for Global Orthopedics and Traumatology (IGOT) in the US and teaching hospital partners in Ghana, Malawi, Nepal, Nicaragua, and Tanzania. The final five manuscripts describe short term educational offerings for physical therapy in Malawi (Beling and Chisati); a short, intensive course on fundamental aspects of clinical research for orthopedic surgeons in Cuba (Miclau et al.); education for midwives in Sudan (Downes et al.) and in Guatemala (Hernandez et al.); and a two day continuing education program for musculoskeletal disorders in Guyana (Ferreira).

As we state in the conclusion of the editorial preceding Part I of this e-book, we “are pleased to be able to share so many creative, interesting, and diverse models of global health initiatives. We applaud the authors for their contributions that demonstrate how educational international partnerships can strengthen health care workforce capacity globally.” The manuscripts from the authors for Part II of this e-book highlight important elements and aspects of global health collaboration. This e-book adds to the body of literature to advance equitable, ethical and sustainable global health partnerships. Collectively the e-book offers strong exemplars of the processes noted by Pinner and Kelly to move equitable partnerships to provide sustainable programs that strengthen health workforce capacity.

## Author contributions

The author confirms being the sole contributor of this work and approved it for publication.

## Conflict of interest statement

The author declares that the research was conducted in the absence of any commercial or financial relationships that could be construed as a potential conflict of interest.
